# Pediatric Prostatic Alveolar Rhabdomyosarcoma Presenting with Metastatic Spinal Cord Compression in the Thoracic Spine: A Case Report and Review of the Literature

**DOI:** 10.7759/cureus.56547

**Published:** 2024-03-20

**Authors:** Matthew T Perry, Andrew J Witten, Majeed Marwan, Alexander Vortmeyer, Jignesh Tailor

**Affiliations:** 1 Neurological Surgery, Indiana University School of Medicine, Indianapolis, USA; 2 Pathology and Laboratory Medicine, Indiana University School of Medicine, Indianapolis, USA

**Keywords:** pediatric genitourinary oncology, pediatric spine oncology, pediatric hematology-oncology, thoracic spine, alveolar rhabdomyosarcoma, pediatric

## Abstract

Rhabdomyosarcoma (RMS) is a pediatric malignancy with a variable prognosis depending on tumor stage and genotype. There has been a significant improvement in survival rates over the past decades. However, aggressive variants can metastasize to locations that are difficult to treat. We report a case of prostatic alveolar rhabdomyosarcoma with metastases to the bone marrow and thoracic spine in a child. The patient was treated with a multimodal approach that included surgical resection of the epidural mass; the administration of vincristine, dactinomycin, and cyclophosphamide; and radiotherapy. Unfortunately, after six months, the patient showed disease progression and was started on secondary-line treatment. This case illustrates the difficulties in managing end-stage metastatic rhabdomyosarcoma and is the first report of prostatic rhabdomyosarcoma presenting with spinal cord compression in a child.

## Introduction

Rhabdomyosarcoma (RMS) is the most common soft-tissue sarcoma of childhood [1]. Pediatric genitourinary rhabdomyosarcoma (GU-RMS) accounts for 25% of all pediatric soft tissue sarcomas and requires multimodal therapy for management [2]. About 14% of children with RMS will have metastatic diseases at the initial diagnosis, associated with a 5-year survival rate of 20% [1]. It has been shown that about 3-5% of children with primary tumors will develop malignant spinal cord compression, with soft tissue sarcomas being the most common cause in children over five years of age [3-5]. Here, we present a unique presentation of a 14-year-old boy who was found to have metastatic alveolar RMS of prostatic origin with bone marrow and epidural metastases with concomitant spinal cord compression.

## Case presentation

Preoperative course

A 14-year-old male with a past medical history of hypospadias initially presented with a syncopal episode two days prior and vague pain in his bilateral lower ribs, bilateral upper abdominal quadrants, left hip, left thigh, and back for the previous two weeks. Additionally, he endorsed urinary retention for the past four weeks, necessitating multiple emergency department visits for intermittent catheterizations and outpatient urology workups. Upon physical examination, the patient exhibited tenderness at the sites mentioned above and bilateral lower extremity hyperreflexia. Strength and sensation were intact in the bilateral lower extremities. Baseline lab work revealed anemia (hemoglobin 10.8 GM/dL) and thrombocytopenia (58 k/cumm), raising concern for potential malignancy or systemic disease. The continued workup revealed concomitant hyperphosphatemia (10.8 mg/dL), hyperuricemia (12.0 mg/dL), and elevated lactate dehydrogenase (LDH) (3641 units/L). A summary of the pertinent laboratory findings can be found in Table 1.

**Table 1:** Clinically significant findings in the patient's initial laboratory workup

Laboratory test	Lab results	Normal/reference range value
WBC count	4.5 k/cumm	3/6-10.6 k/cumm
Hemoglobin	10.8 GM/dL	13.4-17.0 GM/dL
Platelet count	58 k/cumm	150-450 k/cumm
Prothrombin time (PT)	15.4 seconds	<14.1 seconds
International normalized ratio (INR)	1.29	<1.17
Activated partial thromboplastin time (aPTT)	25.5 seconds	<38.4 seconds
Phosphorus	5.8 GM/dL	2.6-5.2 GM/dL
Calcium	10.8 GM/dL	8.5-10.5 GM/dL
Uric acid	12.8 GM/dL	3.0-.8.0 GM/dL
Lactate dehydrogenase	3641 units/L	140-271 units/L

**Figure 1:**
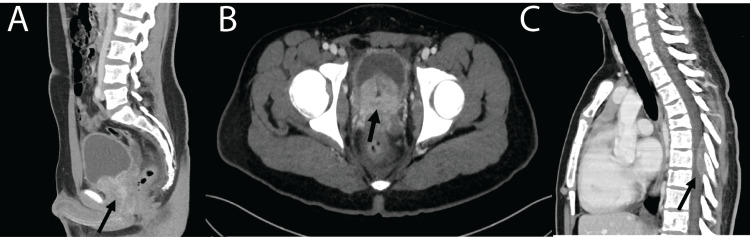
Initial non-contrast CT of the abdomen and pelvis A and B: Axial and sagittal non-contrast CT imaging demonstrating a large prostatic mass (black arrow) infiltrating the base of the bladder; C: Sagittal non-contrast CT of the chest demonstrating compression deformities of T4 and T5 along with intraspinal metastasis spanning from T6 to T8 (black arrow).

**Figure 2:**
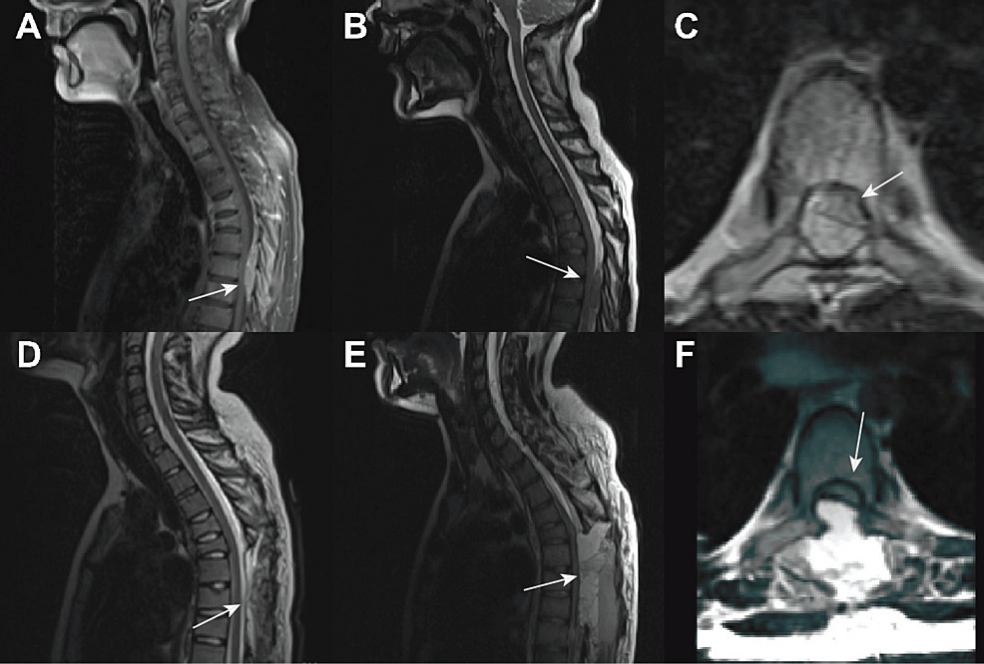
Pre and postoperative MRI A: Sagittal T1 with contrast MRI scan showing epidural intramedullary metastasis in the thoracic level six through eight; B: Sagittal T2 MRI scan showing epidural extramedullary metastasis in thoracic level six through eight; C: Axial T1 with contrast MRI scan showing thoracic epidural extramedullary metastasis D: Postoperative day 1 sagittal T2 MRI scan detailing the thoracic level six through eight laminectomies and decompression; E: Postoperative day 9 sagittal T2 MRI scan detailing the new acute onset thoracic epidural hematoma; F: Postoperative day 9 axial T2 MRI scan detailing the new acute onset thoracic epidural hematoma and notable thecal sac compression.

This raised the concern for new-onset malignancy with secondary tumor lysis syndrome and the question of disseminated intravascular coagulation (DIC). The tumor lysis syndrome was managed with rasburicase, and the patient underwent a bone marrow biopsy, revealing non-hematologic malignant cells, suggesting a metastatic solid tumor. Given the concern for malignancy, comprehensive CT scans were ordered; they revealed a prostate mass that infiltrated the bladder with subsequent metastases to the pelvis and spine (Figure 1). This was further clarified with an MRI of the spine, which confirmed diffuse bone marrow involvement and a 5 cm hypercellular mass spanning spinal segments T4 to T8. There was extramedullary extradural spinal cord compression and extension of the mass through the T7-T8 neural foramina. Severe Bilsky grade 3 spinal canal stenosis at the T7 level, and pathologic compression fractures of the T4, T5, and T6 vertebral bodies were also noted (Figure 2, A to C) [6]. Considering the severity of the spinal cord compression and the acute onset of myelopathy symptoms, an immediate decompressive resection of the spinal mass was pursued via T6-T8 thoracic laminectomy with epidural tumor resection.

Operative description

The patient was infused with three units of platelets, raising his preoperative platelets to 85 k/cumm. Neuromonitoring was initiated for his bilateral lower extremities, capturing somatosensory evoked potentials (SSEPs) and motor-evoked potentials (MEPs), and baseline measurements were established before positioning. He was then carefully positioned in a prone orientation and prepped in a standard fashion. A T6-T8 laminectomy exposed the tumor. The yellow ligaments were incised, allowing the tumor to be rolled out of the left-sided gutter to expose the dura. From there, a plane developed between the dura and the tumor. The tumor was not adherent to the dura but did adhere to the ligamentous complexes in the lateral recess gutters. Following the previously established plane, the tumor was extracted. Intraoperative frozen pathology was consistent with malignancy. All additional tumor samples were sent for permanent pathological analysis. With about 200 mL of estimated blood loss and preoperative anemia/thrombocytopenia, the patient was transfused with one unit each of packed red blood cells and platelets. A Hemovac® (Zimmer Biomet, Warsaw, IN, USA) drain was placed, and the wound was closed with Vicryl® (Ethicon Inc., Cincinnati, OH, USA) and running nylon due to the expectation of adjuvant therapy for the malignancy. The SSEPs and MEPs remained present, reproducible, and comparable to the baseline throughout the procedure. The patient remained stable throughout the operation. Postoperatively, the patient was extubated and transferred to the pediatric ICU (PICU) for close observation and pain management.

Postoperative course

The patient did well in the initial postoperative period and resumed his vincristine-actinomycin D-cyclophosphamide (VAC) regimen on postoperative day (POD) 4. Unfortunately, on POD 9, he developed acute lower extremity weakness and was found to have spinal epidural hematoma, which necessitated a return to the operating room twice for acute hematoma evacuation, which was likely associated with coagulopathy secondary to severe disseminated intravascular coagulation (DIC) and systemic disease. The patient’s lower extremity strength did not improve in function. The patient went on to receive a total of nine cycles of VAC therapy and radiation therapy. Unfortunately, repeat imaging (Figure 2, D to F) revealed disease progression with several new osseous foci in his lower extremities and spin. He was started on ifosfamide/doxorubicin therapy and received two cycles.

Pathologic findings

**Figure 3:**
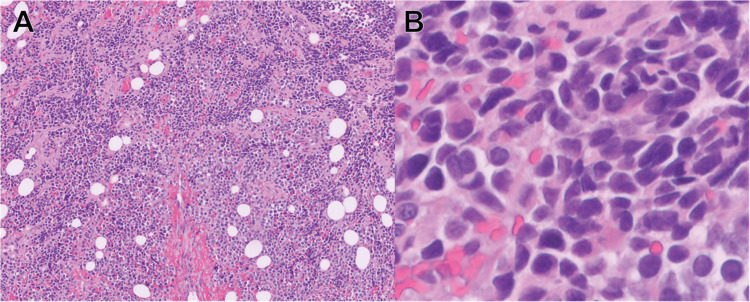
H&E stains from surgical pathology A: H&E (10x) section showing small round blue cell neoplasm; B: H&E (80x) section showing cells that are round or polygonal H&E: Hematoxylin & eosin

**Figure 4:**
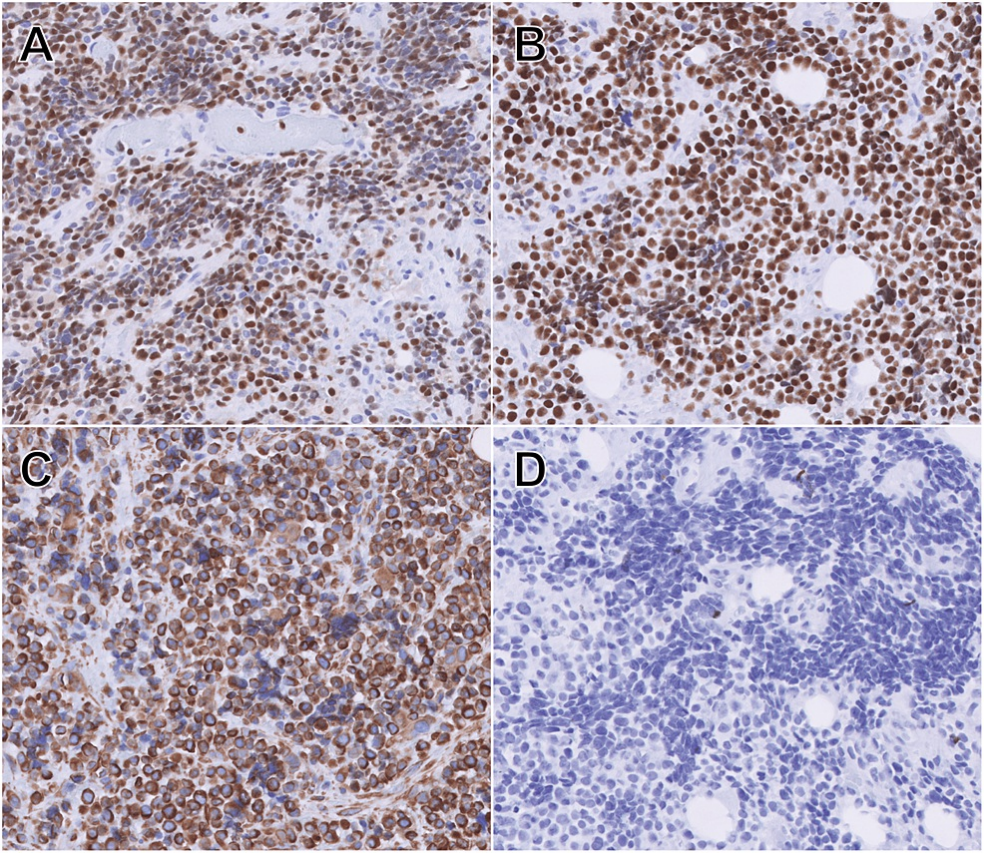
Immunohistochemistry of surgical pathology demonstrating tumor cells that are positive for myogenin (A), MyoD1 (B), and vimentin (C), and negative for CKAE1/AE3 (D).

Sections revealed poorly differentiated small tumor cells with nests of round tumor cells, some of which displayed clear cytoplasm (Figure 3). Occasionally seen were round or polygonal cells with eosinophilic cytoplasm; strap cells were not obvious. Rare tumor cells are large, multinucleated, and have eosinophilic cytoplasm. The tumor cell clusters are separated by fibrous septae. Staining for desmin, actin-HH35, and actin-A14 highlighted rare scattered tumor cells. Myo-D1, myogenin, and vimentin were positive in the tumor cells. The CK AE1/AE3, CAM5.2, and myoglobin were negative in the tumor cells (Figure 4). The tumor phenotype and immunohistochemistry profile were most consistent with alveolar RMS. The cytogenic analysis identified a pathogenic gene variant of TP53 and the pathogenic fusion protein PAX3. These results are also most consistent with a diagnosis of alveolar RMS. 

## Discussion

Rhabdomyosarcoma is a complex disease that can originate in nearly any organ and accounts for 5% of pediatric cancers [1,7]. Genitourinary rhabdomyosarcoma accounts for 25% of all RMS in the pediatric population, with the most common origin sites being the prostate, bladder, and paratesticular regions, followed by the vagina and uterus [8]. There is a bimodal age distribution, with the first and peak incidence being in the first two years of life and the second in adolescence [7]. There is a slight male predominance, with a male-to-female ratio of 1.5:1 [8]. The disease is mostly sporadic but has been observed in several genetic syndromes, including but not limited to Li-Fraumeni syndrome, neurofibromatosis type I, and Costello syndrome [8]. Prostatic RMS is characterized by rapid progression and is typically found to have extensive periurethral, perivesical, and perirectal invasion [9]. This leads to the most common presenting symptoms of urinary retention: urgency, frequency, incontinence, constipation, hematuria, and abdominal mass [7,8].

There are three pathologic subtypes of RMS: embryonal, alveolar, and undifferentiated. Embryonal RMS accounts for 80% of all cases, and alveolar RMS accounts for 15% to 20%, with the rest being undifferentiated [8]. Of the subtypes, alveolar and undifferentiated RMS carry the poorest prognoses. Recent molecular studies of alveolar RMS demonstrated that balanced chromosomal translocations are present 80% of the time and result in the creation of fusion proteins (PAX3-FOX01 or PAX7-FOX01). These proteins are transcription factors, and their fusion induces cell transformation, myogenic differentiation, and apoptosis inhibition. They are thought to confer the aggressive phenotype seen in the alveolar RMS [8]. The tumor is known to disseminate widely to the most common metastatic sites, including the lung, bone marrow, and bone, as seen in our patient [1,9].

Around 14% of children with RMS are found to have metastatic disease at the time of initial diagnosis [1]. The three-year overall survival rate for these patients is 34% [10]. Of the patients with metastatic RMS, 22% have metastasis to their bone marrow, and RMS with bone marrow metastasis accounts for 6% of all RMS cases [1,10]. Our patient reflects what has been previously documented in the literature by Bailey and Wexler: age ≥ 10 years old, diagnosed with alveolar RMS, and ≥3 sites of metastasis [10]. Although rare, an association has been previously described in the literature between RMS presenting with clinical or laboratory features of DIC and tumor lysis syndrome. These presentations were mainly seen in widespread alveolar RMS metastatic to bone marrow [11]. From what has been described in the literature to this date, the patient’s combination of alveolar RMS who are PAX3-FOX01-positive with widespread metastasis involving the bone marrow and features of tumor lysis syndrome and DIC portends to very poor outcomes and low three-year overall survival rates [10-12].

The treatment of RMS varies depending on staging, location of the initial tumor, and pathologic subtype [7,9]. Management has changed significantly over the past few decades. It is generally thought that a multimodal approach involving multiagent chemotherapy, radiotherapy, and radical surgery with goals of organ preservation is best, and overall outcomes have correspondingly improved [2,8]. The current gold standard for chemotherapy treatment is VAC, as established by the Intergroup Rhabdomyosarcoma Study Group (IRSG) and Children’s Oncology Group studies [2].

Our patient presented with a 5 cm epidural mass with Bilsky grade 3 compression at the level of T7. It is estimated that 4% to 25% of patients with systemic malignancy will develop spinal cord compression at some point in time during their disease course, with the most common cause being an extradural tumor [4,13,14]. Given the patient’s presentation, the uncertainty of diagnosis, and the severity of spinal cord compression, surgical decompression was pursued to prevent further neurologic compromise. The patient’s difficulty with maintaining hemostasis postoperatively is likely explained by the fact that DIC in malignancy is often more temperate than its presentation in sepsis and trauma [15]. Studies have indicated that solid tumor cells and injured endothelial cells release procoagulant factors. However, this does not always result in overt symptoms and instead reflects a picture of chronic or low-grade DIC [11]. Careful management of hemostasis is required to prevent complications such as the one seen with our patient.

Our case delineates a unique presentation of RMS, emphasizing its rarity in children with a prostatic primary source. While RMS is known to be diverse in its origins, its manifestation in the prostate, particularly in pediatric patients, is exceptionally rare. Our patient's clinical course, characterized by the combination of alveolar RMS that is PAX3-FOX01-positive, widespread metastasis to the bone marrow, and concomitant features of tumor lysis syndrome and DIC, is an unprecedented constellation of findings in pediatric patients with RMS. Such unique combinations accentuate the unpredictable nature of RMS and stress the importance of sharing such experiences for future diagnostic and therapeutic reference.

This report not only contributes to the limited knowledge base about such uncommon presentations but also encourages a multidisciplinary approach to managing RMS, which is vital given the rapid progression and potential complications associated with this malignancy. Our hope is that this report serves as a foundation for further investigations and discussions and a source of reference for clinicians who may encounter similar rare presentations of RMS in their practice.

## Conclusions

This case illustrates the difficulties associated with the management of pediatric patients with RMS, including diagnosis and surgical management. Our patient with prostatic alveolar RMS presented with spinal cord compression from epidural spinal metastases and bone marrow failure. Management of these patients is challenging due to the risks associated with coagulopathy and the aggressive course of malignancy.
